# Wavelength Tuning in Resonant Cavity Interband Cascade Light Emitting Diodes (RCICLEDs) via Post Growth Cavity Length Adjustment

**DOI:** 10.3390/s24123843

**Published:** 2024-06-14

**Authors:** Nicolas Schäfer, Robert Weih, Julian Scheuermann, Florian Rothmayr, Johannes Koeth, Sven Höfling

**Affiliations:** 1 nanoplus Advanced Photonics Gerbrunn GmbH, 97218 Gerbrunn, Germany; 2 Julius-Maximilians-Universität Würzburg, Physikalisches Institut, Lehrstuhl für Technische Physik, 97074 Würzburg, Germany

**Keywords:** resonant cavity interband cascade light emitting diodes, gas sensing, mid-infrared, distributed Bragg reflector, microcavity

## Abstract

We demonstrate substrate-emitting resonant cavity interband cascade light emitting diodes (RCICLEDs) based on a single distributed Bragg reflector (DBR). These devices operate in continuous wave mode at room temperature. Compared to standard ICLEDs without a cavity, we achieved an 89% reduction in the emission spectrum width, as indicated by the Full Width Half Maximum (FWHM) of 70 nm. Furthermore, we observed far-field narrowing and improved thermal stability. A single DBR configuration allows the cavity length to be adjusted by adding refractive index-matched material to the top of the epitaxial structure after epitaxial growth. This modification effectively shifts the cavity response towards longer wavelengths. We fabricated emitters comprising two cavities of different lengths, resulting in the emission of two distinct spectral lines that can be independently controlled. This dual-color capability enables one of the emission lines to serve as a built-in reference channel, making these LEDs highly suitable for cost-effective gas-sensing applications.

## 1. Introduction

Technical advancements in the fields of chemical sensing, infrared scene projection, industrial process control, and spectroscopy have led to a growing demand for light sources that emit in the mid-infrared (MIR) wavelength band, spanning from ~2 to 20 µm. The three different key technologies for efficient MIR light emission are lasers, LEDs, and thermal emitters. Interband cascade lasers have gained prominence in recent times as an important source primarily for trace gas sensing, owing to their continuous wave operability and low threshold currents, e.g., [1,2,3,4]. However, these lasers are complex devices which can be costly to manufacture and implement. For many applications, mid-infrared LEDs offer an attractive and cost-effective alternative. In comparison to thermal sources, LEDs are more robust and can be operated at high modulation rates, resulting in lower average power consumption. Additionally, LEDs are not subject to the same restrictions that may apply to thermal emitters due to the high temperatures involved, making them more suitable for portable instruments.

ICLEDs derive spontaneous emission from band-to-band electron-hole recombination with multiple cascading stages to recycle the electrons. This design enables a single electron to generate multiple photons, resulting in substantially higher output power and quantum efficiency compared to conventional LEDs operating in the mid- and far-infrared spectral range. Moreover, the compressively strained type-II quantum wells used in ICLEDs are known to suppress Auger recombination losses [5]. This suppression positively influences the output power, temperature stability, and linearity of these devices.

A maximum output power of 5.1 mW and a wall plug efficiency (WPE) of 0.7% at ambient temperature [6] has been achieved following recent advances such as stage-positioning [7] and high-transparency substrates for flip-chip mounted devices. These two factors are essential in reducing internal optical losses, making ICLEDs excellent light sources for spectroscopic applications [8]. While the spectral linewidth of ICLEDs can be partially controlled through stage-positioning, the resulting emission spectrum tends to be relatively broad. Although this broad spectrum can be advantageous for various applications, it inevitably leads to lower spectral power densities. Additionally, extracting the isotropically emitted photons is primarily limited by Fresnel reflection and total internal reflection, which have a substantial effect because of the high refractive index materials. It is therefore desirable to achieve a greater control over the optical mode. One promising approach is to exploit microcavity effects that arise when placing the optical active region within a Fabry–Perot cavity [9].

Resonant cavity LED (RCLED) effects have been widely demonstrated in both the visible and MIR region, showcasing several advantages over conventional LEDs including narrow linewidth, improved directionality, high brightness, and high efficiency [10,11,12,13]. Typically, RCLEDs consist of an optically active region enclosed between two distributed Bragg reflectors, forming a Fabry–Perot cavity in top-emitting devices. However, the epitaxial growth of AlAsSb/GaSb DBR pairs for the MIR spectral region is time consuming and their additional electrical resistance can induce self-heating of the devices, triggering non-radiative recombination. The additional voltage drop limits the overall efficiency of the devices [11]. This is particularly true for the bottom DBR, where more than 20 Bragg pairs are typically required to achieve high reflectivity. Commonly used designs necessitate an annular anode contact pad for current injection, leading to a radial inhomogeneity. Although the lateral conductivity within the quantum wells of the active region is expected to be high in ICLEDs and the current is expected to propagate uniformly after a certain number of stages, the current density may not be uniformly distributed in the early stages.

We introduce a novel design approach for MIR RCLEDs that utilizes a single DBR as the cathode-side mirror and a highly doped contact layer between the active area and the DBR. In this configuration, the anode DBR is replaced by a metal mirror, resulting in a substrate-emitting device that necessitates flip-chip mounting. The concept of employing intracavity contacts has its origins in the VCSEL development and has been successfully adapted to RCLEDs [13]. This arrangement effectively eliminates any current pumping through the DBR and consequently prevents an elevated voltage drop. The metal mirror also acts as a p-side contact, ensuring uniform electrical pumping across the stack. It covers the entire top of the active region to achieve homogeneous current distribution. By adding (or removing) material to (from) the top of the epitaxial structure after growth, this design allows the cavity length to be modified.

## 2. Materials and Methods

### 2.1. Emission Rate Enhancement

Enhanced emission can be achieved by increasing either the spontaneous emission or the excitation rate intensity. The spontaneous radiative transition rate in an optically active, homogeneous medium is given by:

(1) $$W_{spont}=\tau_{spont\;}^{-1}=\int_{0}^{\infty }W_{spont}^{\left(l\right)}\;*\;\;\rho \left(\nu_{l}\right)*\;d\nu_{l}.$$

where $W_{spont}^{\left(l\right)}$ represents the spontaneous radiative transition rate into the optical mode l. $\rho \left(\nu_{l}\right)$ is the mode density and $\tau_{spont}$ is the spontaneous lifetime. The spontaneous emission of an optically active medium can be influenced by tailoring its photonic environment. In RCLEDs, positioning the optically active medium within a micro cavity strongly influences the optical mode density $\rho \left(\nu_{l}\right)\;$ depending on the direction in respect to the Fabry–Perot cavity. This alteration enhances the interaction between the emitted photons and the surrounding optical modes, leading to increased spontaneous emission at the resonance wavelength. The emission rate enhancement at the resonance wavelength is determined by the ratio between the cavity mode density and the free space mode density, which in one dimension and for large $R_{1}$ and $R_{2}$ can be expressed as [14]:

(2) $$G_{e}=\frac{\rho_{cavity}}{\rho_{freespace}}\approx \;\frac{2{\xi \left(R_{1}R_{2}\right)}^{\frac{1}{4}}\left(1-R_{1}\right)}{{\left(1-\sqrt{R_{1}R_{2}}\right)}^{2}}\cdot \frac{\tau_{cav}}{\tau }$$

where $R_{1}$ is the reflectivity of the bottom DBR and $R_{2}$ is the reflectivity of the top contact metallization. The term ξ, denoting the antinode enhancement factor, is equal to 2 because the active region is precisely located at an antinode of the optical field inside the cavity. The ratio between the carrier lifetime with a cavity and the lifetime without a cavity $\tau_{cav}$/τ ≥ 0.9 [15] is typically close to one. This ensures that the presence of the cavity does not significantly alter the overall carrier lifetime. However, the distribution of emitted photons can shift towards optical modes that are resonant with the cavity, which is often referred to as “mode filtering” or “mode selection”.

**Figure 1:**
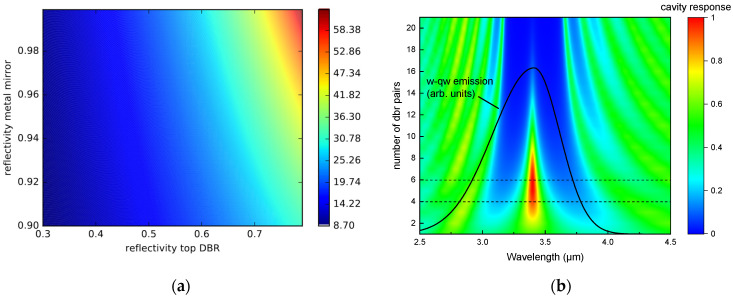
Simulated emission rate enhancement as a function of the mirror and DBR reflectivity (**a**) and spectral cavity response for a single-sided DBR a cavity order $m_{c}=2$ (**b**).

Figure 1a illustrates the emission rate enhancement as a function of the mirror and DBR reflectivity. Optimal results are obtained when $R_{2}$ and $R_{1}$ are close to 1. Achieving such high reflectivity with a metallic mirror can be achieved easily, e.g., by utilizing a SiN/Ti/Ag layer sequence, resulting in $R_{2}$ > 0.94 across the entire spectral range. However, implementing a DBR mirror with similarly high reflectivity would be impractical due to the large number of required layer pairs, resulting in a thick overall layer structure and significantly extended epitaxial growth times. Nevertheless, even with relatively low DBR reflectivities of $R_{1}$ = 0.3, the spontaneous emission rate into the optical mode experiences an eight-fold increase.

The total enhancement integrated over the entire wavelength range can be described as [14]:

(3) $$G_{int}=G_{e}\cdot \sqrt{\pi ln2}\cdot \frac{\Delta \lambda_{cav}}{\Delta \lambda_{ref}}$$

and considers that the off-resonance emission is suppressed, resulting in a narrow linewidth of the emission spectrum. The integrated enhancement does not necessarily have to be >1 to realize a useful RCLED. A crucial factor to consider here is the wavelength range of interest. If the interesting range falls within the resonance peak of the microcavity, $G_{e}$, the optical mode density can be orders of magnitude higher than the free space optical mode density, even with a lower total emission integrated over the entire emission range.

Figure 1b shows the spectral cavity response for different numbers of DBR pairs and a cavity order $m_{c}=2n\left(L=\frac{\lambda }{n}\right)/\lambda =2$, calculated using the Transfer Matrix Method. The black line represents the intrinsic emission spectrum of the optically active region, obtained from measurements of an ICLED grown under identical conditions and recipe, but without the DBR mirror.

As the number of DBR pairs increases, the stopband narrows, causing the first-order maxima of the cavity response to shift towards the center of the intrinsic emission spectrum, which results in prominent side peaks alongside the main peak. To achieve a single peak emission without significant side peaks, we limited the number of DBR layer pairs to 6 for the RCICLED (dashed lines). There is a trade-off between high emission enhancement and effective suppression of side peaks.

### 2.2. RCICLED Design, Growth, and Fabrication

In general, a lower cavity order *m_c_* increases the overlap between the underlying emission spectrum and the cavity response, leading to improved extraction efficiency [16]. However, from a processing perspective, certain constraints such as etch depth tolerances and diffusion of the contact metallization during annealing must be considered. This means that the contacting layer inside the microcavity cannot be made infinitely thin [16]. We have chosen $m_{c}=3$, which allows a sufficiently thick intracavity contact while sacrificing some of the quality factor compared to a lower order cavity.

The DBR mirror was engineered using GaSb (n ≈ 3.76) and AlAsSb (n ≈ 3.16) layers. With the relatively large refractive index contrast (Δn ≈ 0.6), a small number of λ/4 thick layer pairs are sufficient to achieve high reflectivity. Unlike the metallic backside mirror, the electric field can penetrate a certain distance into the DBR. The depth measured from the interface where an ideal mirror interface should be positioned to give rise to the same phase variation is defined as the effective penetration depth. This penetration depth must be added to the cavity thickness to determine the effective cavity thickness $L_{eff}$. Assuming that third- and higher-order terms in r can be neglected, the effective length is [17]:

(4) $$L_{eff}=\frac{1}{2\kappa }\tanh\left(\kappa L_{DBR}\right)$$

$L_{DBR}$ is the total length of the DBR, with the coupling constant given by:

(5) $$\kappa L_{g}=2mr$$

for a single-sided DBR with m DBR pairs. *r* is the reflectivity going from segment 2 into segment 1 with *r* = n2 − 1/(n2 + n1). Given the symmetry conditions, if light were to propagate in the opposite direction, the photons would experience a reflectivity of −r.

**Figure 2:**
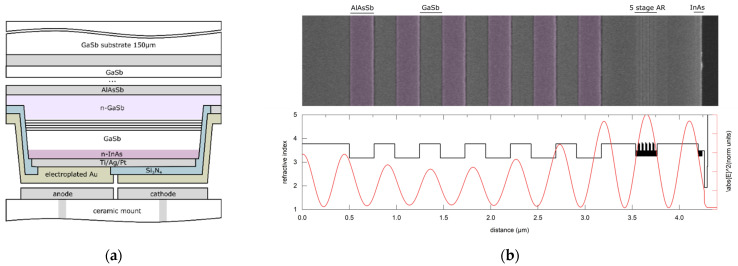
Schematic of a flip-chip mounted RCICLED with a Fabry–Perot cavity formed by a metal mirror and a DBR mirror (**a**). The 5-stage active region is located inside the Fabry–Perot cavity at an antinode of the electric field (**b**).

The layer structure growth using molecular beam epitaxy (MBE) and the fabrication process closely adhere to the procedure outlined in [6], with only relevant differences mentioned here. The cavity is formed by a Ti/Ag/Pt anode-side metal mirror and a cathode-side distributed Bragg reflector composed of 6 GaSb/AlAsSb layer pairs. The metal mirror provides a reflectivity of R > 94% across the entire wavelength range, while the DBR mirror exhibits a reflectivity peak at 3.4 µm (R = 60%). The 5-stage active region is located inside the Fabry–Perot cavity at an antinode of the electric field. This configuration is illustrated in the schematic in Figure 2. The thickness of the GaSb:Te (1 × 10^18^ cm^−3^) contact layer is limited by the cavity length, with a maximum thickness of 366 nm for $m_{c}=3$. A conventional ICLED with an identical number of stages was fabricated as a reference.

The substrate was thinned to 150 µm, and emitters were diced and flip-chip mounted on AlN heat spreaders, which were subsequently soldered to single-sided aluminum circuit boards. This setup ensures efficient heat transfer during continuous wave mode operation. The polished surface did not undergo any further treatment, resulting in lower efficiency compared to the values reported in [6], where the outcoupling efficiency was enhanced by—at that stage unintentional—surface roughening.

## 3. Results

### 3.1. Microcavity Single Mode Extraction

**Figure 3:**
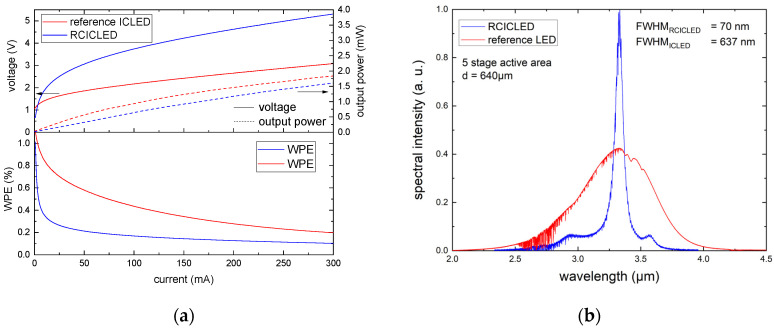
P-I-V characteristics, WPE (**a**) and cw emission spectra of a resonant cavity ICLED and a reference ICLED (**b**) with a mesa diameter of d = 640 µm measured at room temperature.

Figure 3a shows the continuous wave (cw) L-I-V characteristics for both the RCICLED and the reference LED with square-shaped mesa structures (d = 640 µm) at a temperature of T = 20 °C. The total output power was measured using an integrating sphere with a thermoelectrically cooled HgCdTe detector. Auger recombination and lattice heating contribute to the L-I rollover for both devices at high drive currents. The cw output at 200 mA is 1.5 mW for the reference LED and 1.2 mW for the resonant cavity LED with a corresponding voltage drop of 2.8 V and 4.8 V, respectively. The higher voltage drop for the resonant cavity device can be attributed to an excessive etch depth that surpassed the highly doped GaSb layer. The implementation of a grown etch-stop layer could be advantageous in reliably achieving the appropriate etch depth.

The measured V_0_ = 1.8 V is in good agreement with the theoretical value derived from the bandgap energy for each transition E_G_ = 0.37 eV. WPE ranges from ~1% at low drive currents to ~0.3% at 200 mA for the reference LED and a lower 0.5% to 0.2% for the RCLED, mainly due to the higher voltage drop.

Figure 3b showcases the cw emission spectra of the resonant cavity LED and the reference LED with a mesa diameter of d = 640 µm, measured using a Fourier transform infrared (FTIR) spectrometer at room temperature with a drive current of 100 mA. The FWHM for the reference LED is 637 nm with a peak emission at 3.33 µm. The FWHM for the RCICLED is 70 nm at the same peak emission wavelength. This corresponds to a reduction of 89%. Although the total output power over the entire spectral range—which corresponds to the area under the curve—is lower for the resonant cavity device, the intensity at resonant wavelength is 2.3 times higher. The total quantity of photons emitted at a certain frequency is defined as the spectral power density. As mentioned previously, the spectral power density is a crucial device parameter for many applications. In spectroscopy, the relevant measurement window can often be narrow, such as for 3.4 µm methane absorption lines, making a high-power density at this specific window vital. The ratio between the intensity of the main peak and the highest side peaks is 16.

**Figure 4:**
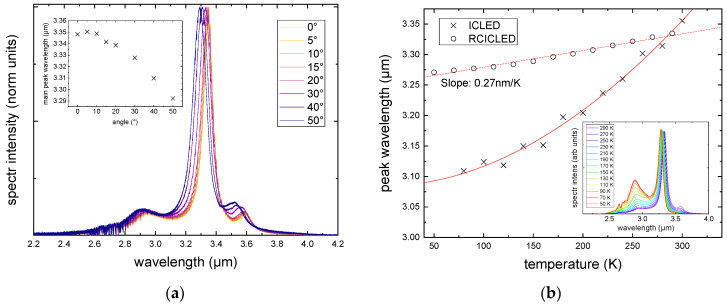
Emission spectra of a RCICLED collected at different angles exhibit a resonance shift to shorter wavelengths when turned away from the optical axis (**a**). Stronger temperature dependence of W-quantum well emission compared to the cavity response (**b**).

Figure 4a presents emission spectra collected at different angles with respect to the optical axis. The collection optics have an opening angle of 56°, allowing for the collection of light within this angular range centered around the optical axis. Photons emitted in a non-vertical direction encounter a detuned DBR due to refraction at the interfaces, leading to changes in the optical path length within the respective layers. Consequently, the resonance shifts to shorter wavelengths for larger emission angles.

The inset in Figure 4b highlights that the underlying emission spectrum of the W-quantum well exhibits a stronger drift with increasing temperature. Therefore, at low temperatures, the high-energy side peak is highly prominent, while at high temperatures, both side peaks become more balanced in height, leading to the highest peak-to-side peak ratio at T = 273 K. The peak shift in the emission of the quantum well and standard LEDs is primarily influenced by the bandgap shift. In contrast, for RCLEDs, the main peak energy shifts 0.27 nm/K due to the temperature-dependent refractive index n(T) of the GaSb buffer layer. The lower drift due to temperature changes is what we refer to as thermal stability. The cavity response is optimized for room temperature operation. The peak wavelength is with and without the cavity overlay at ~290 K.

**Figure 5:**
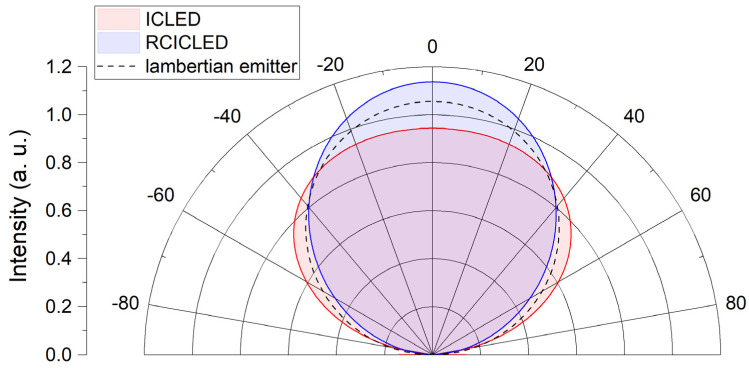
Emission patterns of an ICLED and a RCICLED compared to the theoretical pattern of a perfect Lambertian emitter. Cavity effects only affect the vertically emitted part of the light, resulting in an improved directionality.

Figure 5 shows emission patterns for an ICLED and a RCICLED measured at room temperature and 200 mA drive current (cw). For a spontaneous emitter, we would expect an emission pattern very close to that of a Lambertian emitter, where no specific direction is favored. However, this is not generally applicable to the flip-chip mounted ICLEDs we measured. Interference effects from reflections off the backside metal mirror as well as the surface finish of the LED can both impact the emission pattern. This results in a reduced directionality compared to the Lambert emitter. The RCICLED shows a different behavior: the cavity effects only affect the vertically emitted part of the generated photons. This changes the radiation characteristics, resulting in an improved directionality compared to a Lambertian emitter.

### 3.2. Dual Wavelength Operation in RCICLEDs

**Figure 6:**
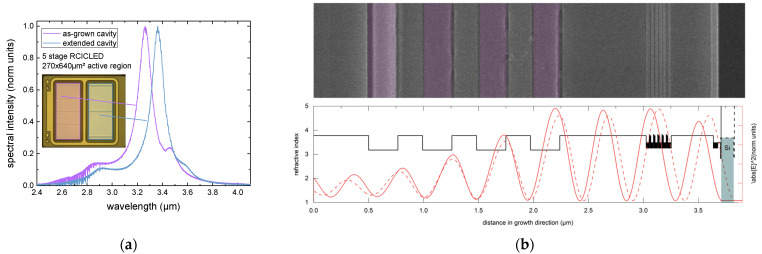
Two spectrally separated peaks of a dual-wavelength RCICLED (**a**). This is achieved by depositing a 120 nm layer of Si on top of the mesa structure after growth (**b**).

In a single DBR configuration, the cavity length can be adjusted by adding refractive index-matched material to the top of the epitaxial structure after growth, thereby shifting the cavity response to a longer wavelength. Using this principle, the peak emission can be tuned to longer wavelengths by varying the amount of material sputtered on top. However, the extent of this wavelength shift is ultimately limited by the spectral emission range of the active stages within the device. The active stages’ emission spectrum defines the maximum and minimum wavelengths that can be effectively achieved through this method. We achieved this wavelength tuning through ion beam sputtering. Specifically, a 120 nm layer of Si was deposited on top of the mesa structure (Figure 6b). Due to a low refractive index contrast between GaSb and Si at the emission wavelength of approximately 3.4 µm (n_GaSb_ = 3.73, n_Si_ = 3.47), no prominent spectral features are expected from the epi stack/Si interface.

To realize dual wavelength RCICLEDs, four AlAsSb/GaSb DBR pairs were grown, resulting in a peak reflectivity of R = 0.36. The cavity order was increased to $m_{c}=4$ to accommodate a thicker intracavity layer of 786 nm. The active stages are located at an antinode of the electric field with respect to the metal mirror. The processing run follows the same procedure as described for the single wavelength RCICLED, with one exception. The 640 × 640 µm^2^ mesa structure was split into two smaller mesa structures with a common cathode contact (inset Figure 6a). The 120 nm Si layer was deposited on only one of the mesa structures. Anode contact metallization was then applied to each structure to individually address both mesa structures.

Figure 6a illustrates the continuous wave spectral emission of each active region. The emission of the as-grown cavity peaks at 3.26 µm, while the extended cavity peaks at 3.36 µm. This results in two distinct peaks. In this setup, each channel is equipped with its own cathode and anode, allowing for independent control of both channels. Consequently, it is possible to drive both wavelengths simultaneously or alternate between them as needed. Additionally, one of the channels can function as a monolithically integrated reference channel in spectroscopy applications. The inset in Figure 6a highlights this modification in the mesa structure and the resulting dual-wavelength emission.

## 4. Discussion

Spectral characterization of RCICLEDs has demonstrated that the distribution of emitted photons shifts towards optical modes that are resonant with the cavity. By replacing one of the DBRs in the conventional RCLED design, we have developed devices with high spectral brightness within a narrow linewidth that are well suited for selective gas detection and optical spectroscopy. To achieve a narrower spectral linewidth and higher optical output at the resonance wavelength, the number of periods in the DBR can be increased. This enhances the cavity finesse and Q-factor. However, it also results in a decrease in the overall output power.

Replacing one of the DBRs with a rear mirror offers several advantages over conventional RCLEDs. They feature a comparatively thinner layer structure since the lower, typically thick DBR is eliminated, reducing growth time, minimizing strain, and lowering the potential for defects. Additionally, they provide improved thermal coupling and lower electrical resistance compared to double-DBR devices [10]. This is facilitated by an intracavity contact layer of highly doped GaSb positioned between the active region and the DBR, allowing only one DBR to be pumped. For flip-chip RCLEDs, the doping in the upper DBR, which is necessary to transport carriers, can induce below-bandgap absorption by free carriers [18,19]. To minimize loss, sections of the DBR where the optical field intensity is low can be highly doped. However, this issue can be completely avoided in single DBR configurations since the upper DBR does not contribute to carrier transport, allowing it to remain undoped.

The emission wavelength is determined by the thickness of the InAs and GaInSb layers in the active quantum well and by the resonant cavity. Spectral measurements shown in Figure 6 indicate that by expanding the cavity through refractive index-matched material, the emission shifts to a longer wavelength. The peak emission can be tuned to longer wavelengths by varying the amount of material sputtered on top, which is a novel approach for mid-infrared emitters.

## 5. Conclusions

In conclusion, this research demonstrates the successful continuous wave operation of RCICLEDs at room temperature. The innovative single DBR configuration, along with the ability to adjust cavity lengths, resulted in substantial improvements in spectral control, thermal tuning, and far-field narrowing compared to standard ICLEDs. The dual-color capability of RCICLEDs, achieved by fabricating emitters with two cavities of different lengths, enhances their suitability for cost-effective gas-sensing applications. The monolithic integration of the dual-color capability also potentially opens up the prospect of mid-infrared LED arrays for dual-color scene projectors.

## Data Availability

The original contributions presented in the study are included in the article, further inquiries can be directed to the corresponding author.
